# Supplementary material for: Phase transitions associated with magnetic-field induced topological orbital momenta in a non-collinear antiferromagnet
Source: Nat Commun. 2024 Jan 27;15:822. doi: 10.1038/s41467-024-45129-x (PMC10821865; doi:10.1038/s41467-024-45129-x)
Supplement: Supplementary file 1 — Supplementary Information [file 41467_2024_45129_MOESM1_ESM.pdf]

SUPPLEMENTARY INFORMATION

Phase transitions associated with magnetic-field induced topological orbital momenta in a non-collinear antiferromagnet

Sihao Deng,[1,2,3,*] Olena Gomonay,[4] Jie Chen,[1, 3] Gerda Fischer,[2] Lunhua He,[3,5 6,*] Cong Wang,[7] Qingzhen Huang,[8] Feiran Shen[1,3], Zhijian Tan[1,3], Rui Zhou[5], Ze Hu,[9] Libor Šmejkal,[4] Jairo Sinova,[4] Wolfgang Wernsdorfer,[2,10] Christoph Sürgers[2, *]

[1] Institute of High Energy Physics, Chinese Academy of Sciences, Beijing 100049, China

[2] Physikalisches Institut, Karlsruhe Institute of Technology, Karlsruhe 76049, Germany

[3] Spallation Neutron Source Science Center, Dongguan 523803, China

[4] Institut für Physik, Johannes Gutenberg Universität Mainz, 55128 Mainz, Germany

[5] Beijing National Laboratory for Condensed Matter Physics, Institute of Physics, Chinese Academy of Sciences, Beijing 100190, China

[6] Songshan Lake Materials Laboratory, Dongguan, Guangdong 523808, China

[7] School of Integrated Circuit Science and Engineering, Beihang University, Beijing 100191, China

[8] NIST Center for Neutron Research, National Institute of Standards and Technology, Gaithersburg, Maryland 20899, USA

[9] Department of Physics and Beijing Key Laboratory of Opto-electronic Functional Materials & Micro-Nano Devices, Renmin University of China, Beijing 100872, China

[10] Institute for Quantum Materials and Technologies, Karlsruhe Institute of Technology, Karlsruhe 76021, Germany

Supplementary Note 1:

Magnetic properties and nuclear magnetic resonance of $Mn_3Zn_{0.5}Ge_{0.5}N$

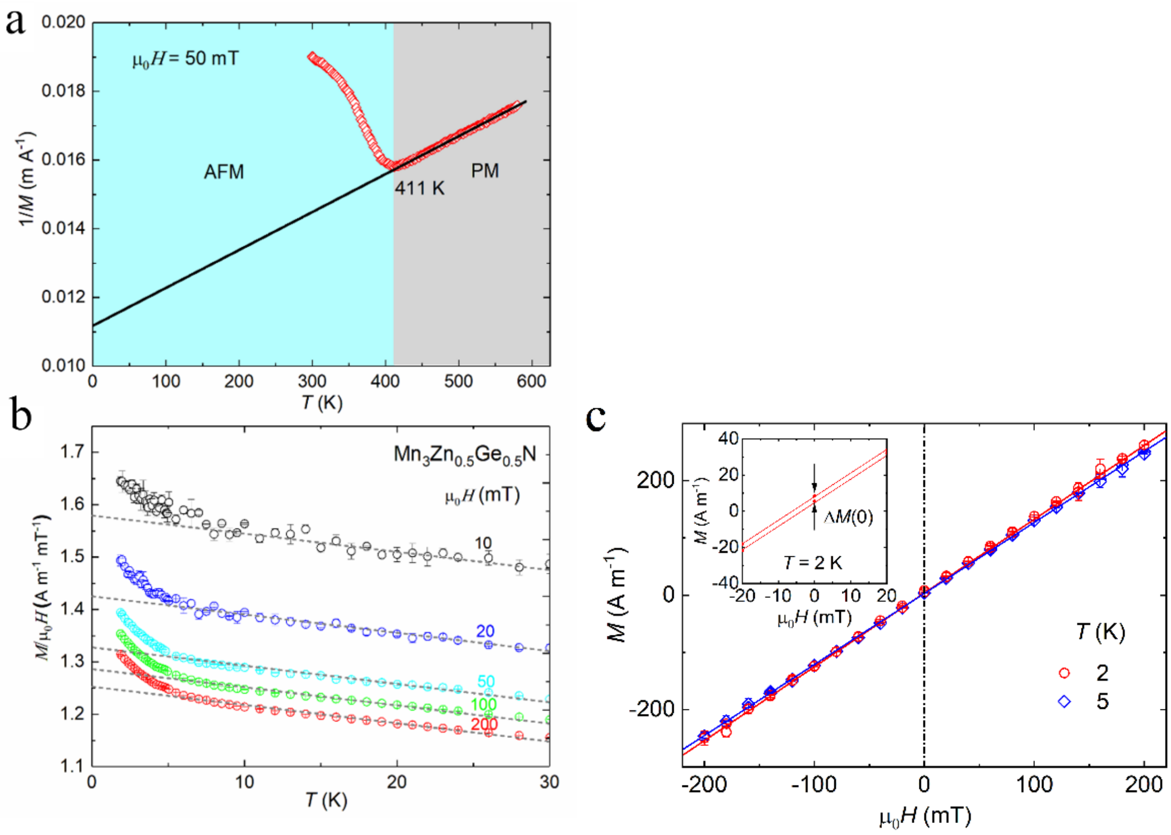

Supplementary Fig. 1 | Magnetic properties of $Mn_3Zn_{0.5}Ge_{0.5}N$. a, Temperature dependence of the inverse of the magnetization *M*. Solid line indicates a linear behavior. Blue (grey) shadowed area indicates the antiferromagnetic AFM (paramagnetic PM) phase. **b**, Temperature dependence of $M/\mu_0 H$ for various *H*. The short-dash lines indicate the linear behavior. **c**, Isothermal magnetization *M*(*H*) at *T* = 2 K and 5 K. Inset shows *M*(*H*) at 2 K in low fields where Δ*M*(0) is the difference of *M*(0) between the up sweep and down sweep of the field *H* . Source data are provided as a Source Data file.

A Néel temperature T_N = 411 K is obtained from the Curie-Weiss behavior $1/M \sim T$ in agreement with previous results[1, 2]. $M/\mu_0 H$ in various fields displays a linear background with a negative slope with an upturn below ~5 K.

The integral magnetization *M* of $Mn_3Zn_{0.5}Ge_{0.5}N$ is small and does not show a transition at low temperatures (Supplementary Fig. 1c). Rather, the static susceptibility $\chi = M/H$ increases linearly toward low temperatures with an upturn below ~ 5 K. The magnetization in 10 mT at T = 2 K corresponds to an average magnetic moment of 3.6×10^{-5} μ_B/Mn. At zero field, a magnetization $M(0) = \Delta M(0)/2$= 1.5 A m^{-1} corresponding to 0.4×10^{-5} μ_B/Mn is verified by

the isothermal magnetization curves at 2 K within our experimental accuracy of 0.3 A m^{-1} (Supplementary Fig. 1d). These tiny magnetizations agree with a very small NMR shift (Supplementary Fig. 2) and the weak linear dependence on *H* confirms that the change of the magnetic structure below T^* = 3.7 K is very small.

The slight change in magnetic properties during cooling is also confirmed by NMR measurements. Zero-field NMR suggests the presence of magnetic order at low temperatures. A variation of magnetic moments of ~1%, would correspond to a shift of the NMR line by ~1 MHz, which cannot be resolved from the measured NMR line with a full width at half maximum of 20 MHz (Supplementary Fig. 2). However, the tiny shift of the NMR peak to higher frequencies at 1.9 K is at least compatible with larger ordered moments at 1.9 K compared to 6.2 K.

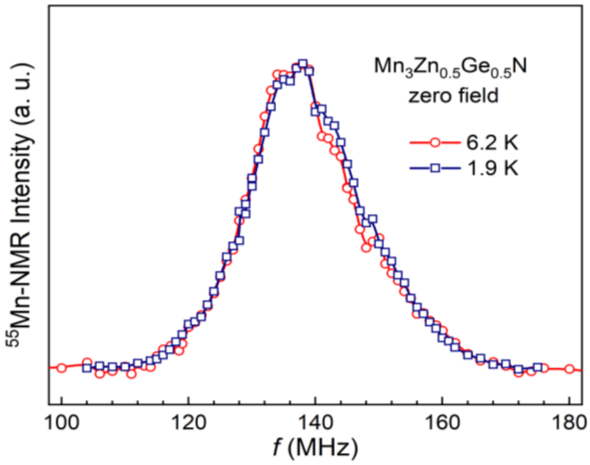

Supplementary Fig. 2 | Nuclear magnetic resonance spectra of ^{55}Mn nuclei in $Mn_3Zn_{0.5}Ge_{0.5}N$. Spectra at temperatures 6.2 K > T^* and 1.9 K < T^*. The data were collected at zero magnetic field. Errors are smaller than the symbol size. Source data are provided as a Source Data file.

Supplementary Note 2:

Electronic transport properties of $Mn_3Zn_{0.5}Ge_{0.5}N$ and $Mn_3Ag_{0.93}N$

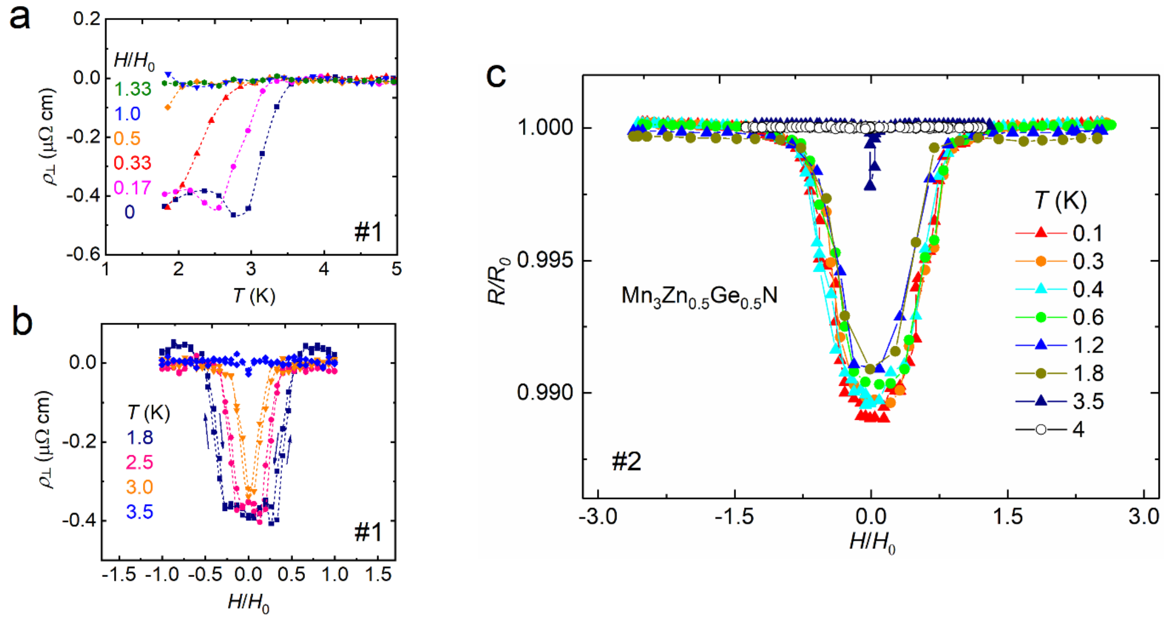

Supplementary Fig. 3 | Transverse resistivities of $Mn_3Zn_{0.5}Ge_{0.5}N$. a, $\rho_\perp(T)$ of sample #1 for various magnetic fields H applied perpendicularly to the current direction. **b,** $\rho_\perp(H)$ of sample #1 in perpendicular magnetic field H at various temperatures T. The transition field H_0 = 30 mT was determined from the resistivity at 1.8 K. The sweep direction of the magnetic field is indicated by arrows. **c,** Field dependence of the transverse resistance ratio R/R_0 for sample #2 at low temperatures, where R_0 is the value at T = 4 K in zero field and H_0 is 120 mT. Source data are provided as a Source Data file.

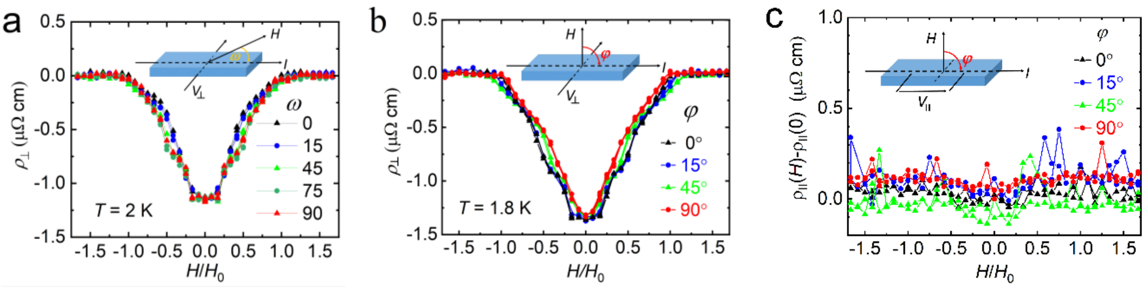

Supplementary Fig. 4 | Angular dependence of resistivity of $Mn_3Zn_{0.5}Ge_{0.5}N$ (#2). a, $\rho_\perp(H)$ for various angles ω between the directions of field H and voltage V at 2 K where H_0 = 120 mT. ω is the angle of in-plane rotation around the surface normal. **b,** Angular dependence of $\rho_\perp(H)$ for various out-of-plane angles φ between the directions of field H and current I at 1.8 K. **c,** Angular dependence of $\rho_{\parallel}(H)$ for various out-of-plane angles φ between the directions of field H and current I at 1.8 K. Source data are provided as a Source Data file.

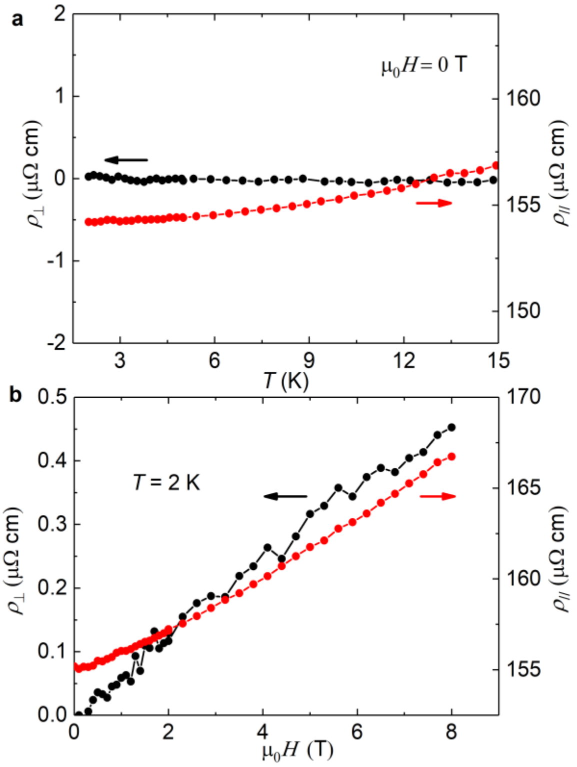

Supplementary Fig. 5 | Resistivity of $Mn_3Ag_{0.93}N$. Longitudinal resistivity and transverse resistivity **a,** vs. temperature and **b,** vs. magnetic field. The slope of $\rho_\perp(H)$ corresponds to a carrier density $n = 1.2 \times 10^{18}$ cm^{-3}. Source data are provided as a Source Data file.

Supplementary Note 3:

Neutron diffraction results of $Mn_3Zn_{0.5}Ge_{0.5}N$

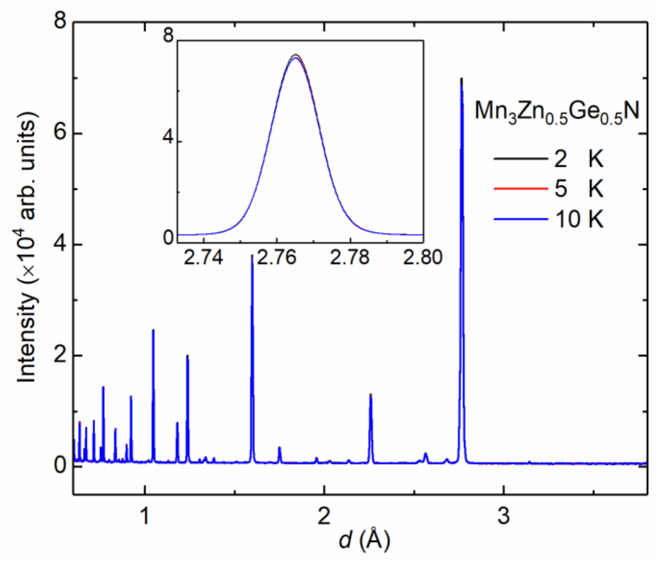

Supplementary Fig. 6 | Neutron diffraction patterns at 2 K, 5 K, and 10 K. The inset shows the reflection around $d = 2.77$ Å. Source data are provided as a Source Data file.

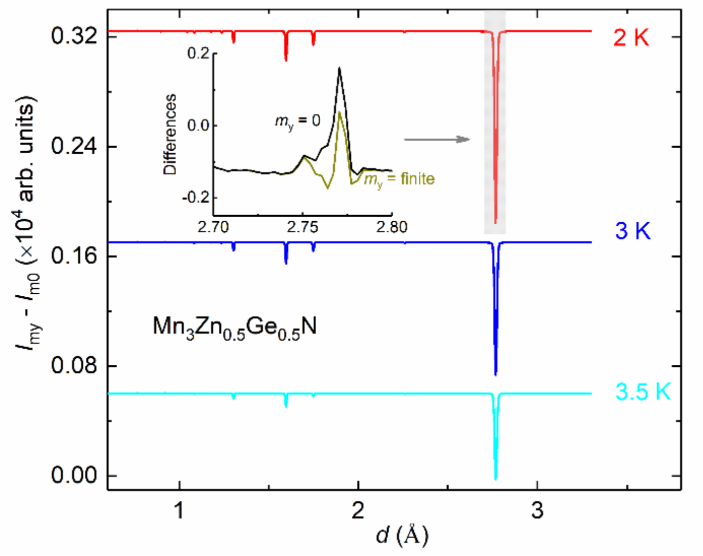

Supplementary Fig. 7 | Intensity difference $I_{my} - I_{m0}$. Data are shown for selected 2 K, 3 K, and 3.5 K where I_{my} and I_{m0} indicate the fitted difference between the observed and calculated intensities of $Mn_3Zn_{0.5}Ge_{0.5}N$ with optimized magnetic moments $m_y = 0.38$ μ_B/Mn or $m_0 = 0$, respectively. Inset shows the difference around $d = 2.77$ Å at 2 K on a smaller scale. Source data are provided as a Source Data file.

Supplementary Table 1 | Refined structural parameters of $Mn_3Zn_{0.54}Ge_{0.46}N$ at 300 K. Space group $Pm\bar{3}m$ (No. 221). Atomic positions: Mn: 3c (0, 1/2, 1/2); Zn/Ge: 1a (0, 0, 0); N: 1b (1/2, 1/2, 1/2).

Atoms and structural parameters		
a (Å)		3.91831(1)
V (Å^3)		60.158(1)
Mn	n	1
Zn	n	0.54(1)
Ge	n	0.46(1)
N	n	1
Phase fraction (wt. %)	$Mn_3Zn_{0.54}Ge_{0.46}N$	96.3
Phase fraction (wt. %)	MnO	3.7
R_p (%)		2.85
R_{wp} (%)		3.78
χ^2		2.611
m_{Mn} (μ_B/Mn)		2.85(3)

a: lattice constant, V: unit-cell volume, n: number of atoms per unit cell, R_p: profile R-factor, R_{wp}: weighted profile R-factor, χ^2: goodness of fit, m_{Mn}: magnetic moment per Mn atom

Supplementary Table 2 | Refined structural parameters of $Mn_3Zn_{0.54}Ge_{0.46}N$ at 10 K. Space group $Pm\bar{3}m$ (No. 221). Atomic positions: Mn: 3c (0, 1/2, 1/2); Zn/Ge: 1a (0, 0, 0); N: 1b (1/2, 1/2, 1/2). The refinement agreement and weighted profile agreement values obtained were R_p = 4.05% and R_{wp} = 6.15%, indicating the high quality of the structural model.

Atoms and structural parameters		
a (Å)		3.91157(2)
V (Å^3)		59.849(1)
Mn	n	1
Zn	n	0.54
Ge	n	0.46
N	n	1
R_p (%)		4.05
R_{wp} (%)		6.15
χ^2		4.164
m_{Mn} (μ_B/Mn)		3.26(3)

a: lattice constant, V: unit-cell volume, n: number of atoms per unit cell, R_p: profile R-factor, R_{wp}: weighted profile R-factor, χ^2: goodness of fit, m_{Mn}: magnetic moment per Mn atom

Supplementary Table 3 | Refined structural parameters of $Mn_3Zn_{0.54}Ge_{0.46}N$ at 2 K for the cases of M_y = 0 and optimized M_y = 0.38 μ_B/Mn. Space group $Pm\bar{3}m$ (No. 221). Atomic positions: Mn: 3c (0, 1/2, 1/2); Zn/Ge: 1a (0, 0, 0); N: 1b (1/2, 1/2, 1/2).

Atoms and structural parameters		M_y = 0 / M_y = 0.38 μ_B/Mn
a (Å)		3.91157
Mn	*n*	1
Zn	*n*	0.54
Ge	*n*	0.46
N	*n*	1
R_p (%)		4.19 / 4.14
R_{wp} (%)		6.42 / 6.40
χ^2		9.252 / 9.190
m_{Mn} (μ_B/Mn)		3.40(2) / 3.32(2)

a: lattice constant, *V*: unit-cell volume, *n*: number of atoms per unit cell, R_p: profile R-factor, R_{wp}: weighted profile R-factor, χ^2: goodness of fit, m_{Mn}: magnetic moment per Mn atom

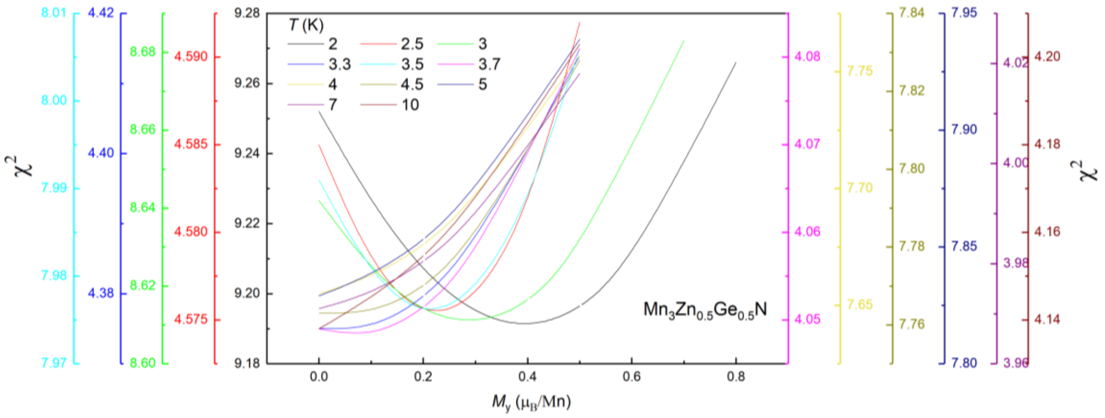

Supplementary Fig. 8 | The magnetic moment M_y dependence of χ^2. χ^2 is determined from the expected and weighted profile *R* factors, and is obtained by fitting the selected M_y. Source data are provided as a Source Data file.

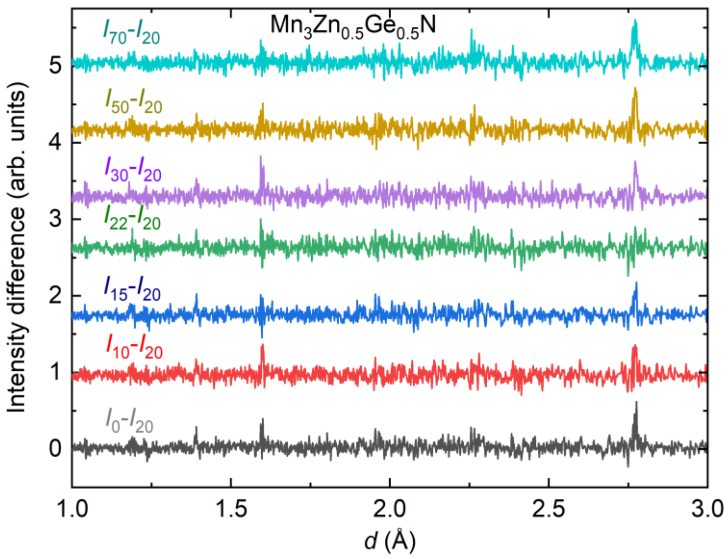

Supplementary Fig. 9 | Intensity difference I–I_{20}=I(μ$_0$$H$)–$I$(20mT) of neutron diffraction patterns. I indicates the intensity under different applied magnetic fields. The offsets are indicated in the image for clarity. Source data are provided as a Source Data file.

Supplementary Note 4:

Neutron diffraction results of isostructural $Mn_3Ag_{0.93}N$

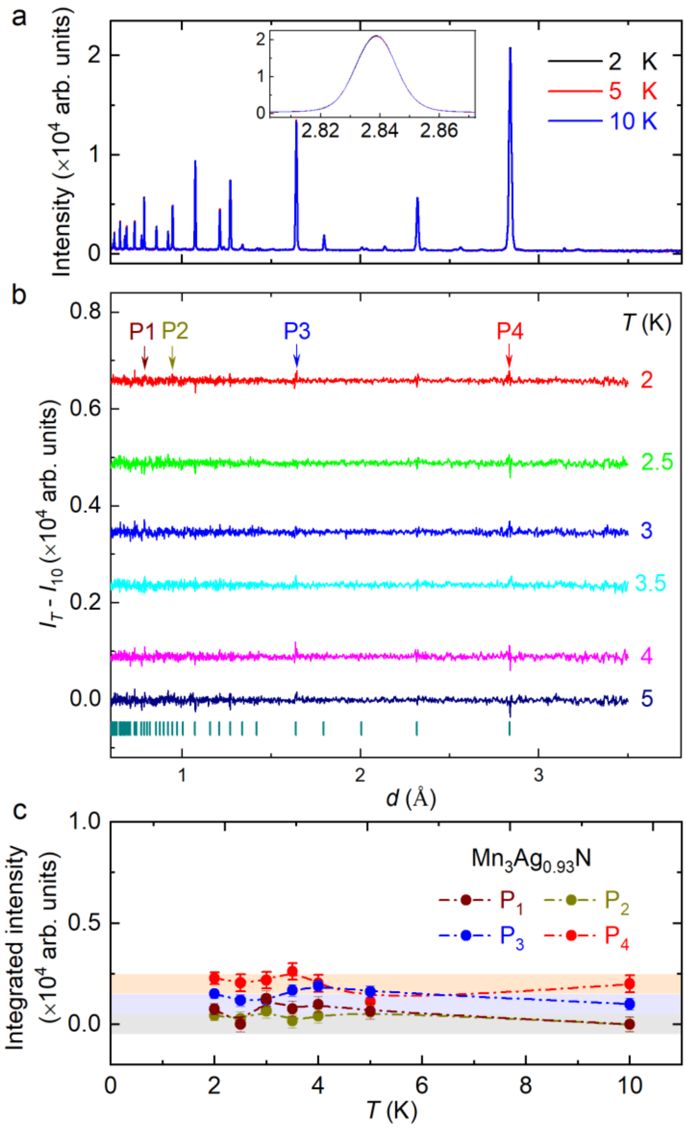

Supplementary Fig. 10 | Neutron diffraction on $Mn_3Ag_{0.93}N$. a, Neutron diffraction patterns at 2 K, 5 K, and 10 K. The inset shows the enlarged peak around *d* = 2.8 Å. **b**, Diffraction patterns of $Mn_3Ag_{0.93}N$ for various temperatures. I_T and I_{10} indicate the intensities of patterns at temperature *T* (K) and 10 K, respectively. The offsets are explored in the image for clarity. **c,** Temperature dependence of the integrated intensity at peak positions P_1 ~ 0.79 Å, P_2 ~ 0.95Å, P_3 ~ 1.64 Å, and P_4 ~ 2.84 Å. The offsets 0, 0, 0.1, and 0.2 were applied for the integrated intensities of P_1, P_2, P_3, and P_4 respectively. Error bars represent the standard deviation. Source data are provided as a Source Data file.

Supplementary Table 4 | Refined structural parameters of $Mn_3Ag_{0.93}N$ at 300 K. Space group: Pm$\bar{3}$m (No. 221). Atomic positions: Mn: 3c (0, 1/2, 1/2); Ag: 1a (0, 0, 0); N: 1b (1/2, 1/2, 1/2).

Atoms and structural parameters		
a (Å)		4.01975(2)
V (Å^3)		64.953(1)
Mn	n	1
Ag	n	0.928(5)
N	n	1
Phase fraction (wt. %)	$Mn_3Ag_{0.93}N$	98.3
Phase fraction (wt. %)	MnO	1.7
R_p (%)		3.66
R_{wp} (%)		4.63
χ^2		2.990
m_{Mn} (μ_B/Mn)		-

a: lattice constant, V: unit-cell volume, n: number of atoms per unit cell, R_p: profile R-factor, R_{wp}: weighted profile R-factor, χ^2: goodness of fit, m_{Mn}: magnetic moment per Mn atom

Supplementary Table 5 | Refined structural parameters of $Mn_3Ag_{0.93}N$ at 2 K. Space group Pm$\bar{3}$m (No. 221). Atomic positions: Mn: 3c (0, 1/2, 1/2); Ag: 1a (0, 0, 0); N: 1b (1/2, 1/2, 1/2).

Atoms and structural parameters		
a (Å)		4.01517(2)
V (Å^3)		64.731(1)
Mn	n	1
Ag	n	0.928
N	n	1
R_p (%)		4.88
R_{wp} (%)		5.99
χ^2		2.306
m_{Mn} (μ_B/Mn)		3.81(3)

a: lattice constant, V: unit-cell volume, n: number of atoms per unit cell, R_p: profile R-factor, R_{wp}: weighted profile R-factor, χ^2: goodness of fit, m_{Mn}: magnetic moment per Mn atom

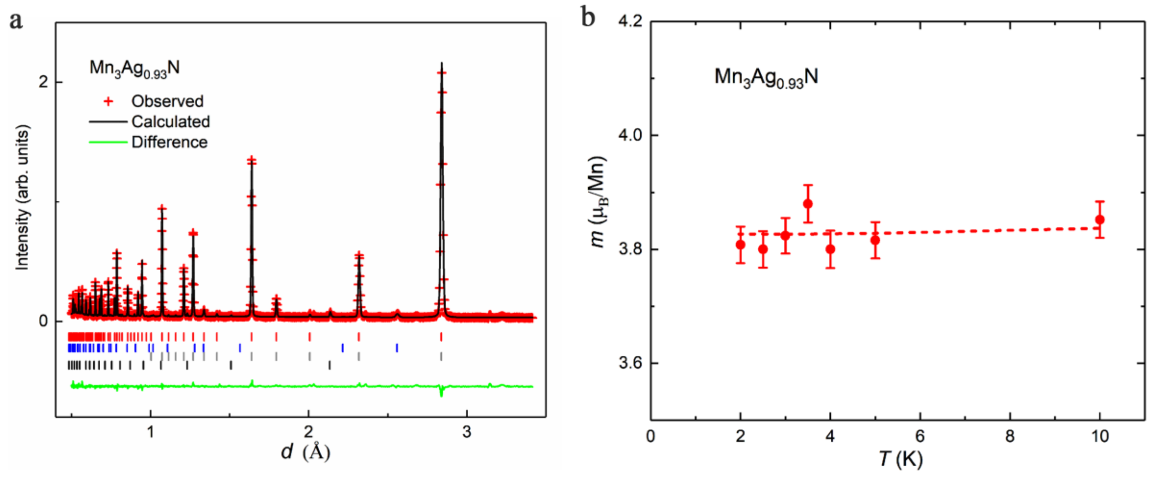

Supplementary Fig. 11 | Powder neutron-diffraction. a, Diffraction pattern at 2 K for the sample of nominal composition $Mn_3Ag_{0.93}N$. The refined composition was determined to be $Mn_3Ag_{0.93}N$. The neutron diffraction pattern are fitted well with the antiperovskite model shown as Fig. 1a. The vertical markers below the data indicate the angular positions of the nuclear (top row), MnO impurity (second row), and magnetic (third row) Bragg reflections. Peaks of vanadium (bottom row) were detected as a vanadium container was adopted to hold samples during the neutron diffraction measurement. The refinement agreement and weighted profile agreement values obtained were R_p = 4.88% and R_{wp} = 5.99%, indicating the high quality of the structural model (Supplementary Table 5). **b,** Temperature dependence of the local magnetic moment of Mn atoms determined by Rietveld refinement of the neutron diffraction data of $Mn_3Ag_{0.93}N$. Error bars represent the standard deviation. Source data are provided as a Source Data file.

Supplementary Note 5:

Theoretical Model

To interpret the observed behavior of the resistivity and the fictional suppression of the magnetization by a magnetic field we first developed a model that describes different antiferromagnetic phases in presence of the external magnetic field $\mathbf{H}$. At the macroscopic level, the magnetic structure of an antiferromagnet $Mn_3Zn_{0.5}Ge_{0.5}N$ is described with three vectors $\mathbf{M_1}$, $\mathbf{M_2}$ and $\mathbf{M_3}$ of magnetic sublattices. Instead, we introduce linear combinations that correspond to the order parameters of different antiferromagnetic phases: two Néel vectors $\mathbf{N_1} = (\mathbf{M_1} + \mathbf{M_2} - 2\mathbf{M_3})/\sqrt{6}$ and $\mathbf{N_2} = (-\mathbf{M_1} + \mathbf{M_2})/\sqrt{2}$, and total magnetization $\mathbf{M} = (\mathbf{M_1} + \mathbf{M_2} + \mathbf{M_3})/\sqrt{3}$.[3,4] Magnetic configurations are calculated by minimizing the free energy of the sample which we model in agreement with the highest spin symmetry group which is isomorphic with D_{3d}:

$$F = \frac{1}{2}J(T)\mathbf{M}^2 + \frac{1}{4}D\mathbf{M}^4 - \frac{1}{2}D'[(\mathbf{N_1}\mathbf{M})^2 + (\mathbf{N_2}\mathbf{M})^2] - \Lambda_{CC}[(\mathbf{N_1}\mathbf{H})^2 + (\mathbf{N_2}\mathbf{H})^2] - \mathbf{MH} \qquad \text{(S1)}$$

Here J stands for the Heisenberg exchange coupling (coupling between pairs of spin), D and D'represent biquadratic exchange couplings (couplings between four spins), and the last term is a standard Zeeman energy in the external magnetic field. The value of the biquadratic exchange couplings (constants D,D') can be calculated from the first principles, as recently demonstrated in Ref. 6. From a phenomenological point of view, the biquadratic exchange stabilizes noncollinear magnetic ordering, as it will be discussed below.

The nontrivial term with the phenomenological constant Λ_{CC} describes the anisotropy of Zeeman interactions associated with the topology of spin ordering (three noncollinear spins). This term is similar to the field-induced anisotropy observed in systems with unquenched orbital momentum (Paschen-Back effect)[5]. In the case of noncollinear antiferromagnets, an orbital momentum appears due to electron hopping between the magnetic atoms, even in absence (or vanishingly small) spin-orbit coupling. A simple explanation of this effect is as follows. A free electron moves in the effective magnetic field induced by exchange interactions

with the localised spins. As the direction of this field rotates from site to site, the electron spin acquires an additional Berry phase.[7] The corresponding effective force on the electron spin is equivalent to the effect produced by spin-orbit interactions. In a coplanar triangular structure, however, both couter-clockwise and clockwise directions of electron motion are equivalent, and the effective orbital momentum is zero on average. In a non-coplanar structure, the out-of-plane localized magnetic moment removes the degeneracy of the couter-clockwise and clockwise electron motions, resulting in a non-zero topological orbital momentum. Interaction between such topological orbital moments according to Ref. 6 is given by the expression $E_{CC} = \kappa_{CC}(\mathbf{M}\cdot\mathbf{N}_1\times\mathbf{N}_2)^2$. If the magnetisation is induced by the magnetic field, then $\mathbf{M} = \chi\mathbf{H}$, where χ is the magnetic susceptibility, and $E_{CC} = \kappa_{CC}\chi^2(\mathbf{H}\cdot\mathbf{N}_1\times\mathbf{N}_2)^2$. The Néel vectors are orthogonal to each other, $\mathbf{N_1}\perp\mathbf{N_2}$ (see below), and thus form an orthogonal frame. As such, we can write $E_{CC} = \kappa_{CC}\chi^2\left[\mathbf{H^2}-(\mathbf{N_1H})^2+(\mathbf{N_2H})^2\right]$. Denoting $\Lambda_{CC} = \kappa_{CC}\chi^2$ brings us to Eq. (1) of the main text.

We calculated different magnetic configurations by minimizing Eq. (1) assuming that (i) the exchange constant $J(T)$ changes with temperature T, and (ii) magnitudes of sublattices magnetizations are equal and fixed: $|\mathbf{M_1}| = |\mathbf{M_2}| = |\mathbf{M_3}| = M_s$.

In absence of the magnetic field (**H**=0), we distinguish between two configurations, Γ^{5g} and FO (Fig. 1 b,d), in which all three vectors sublattice magnetizations are locked within one plane. Configuration Γ^{5g} is pure antiferromagnetic ($\mathbf{M}=\mathbf{0}$), the angle between each pair of sublattice magnetizations is 120°, which means $\mathbf{N_1}\perp\mathbf{N_2}$, $|\mathbf{N_1}| = |\mathbf{N_2}| = \sqrt{3}M_s$. This configuration is stable at $J(T) > 0$. The spin symmetry of the phase is equivalent to the group C_{3d} which includes 120° rotations around the axis perpendicular to the spin plane, and 180° rotation around the axis which is parallel to one of sublattice magnetizations.

Configuration FO (Fig. 1 d) has a nonzero magnetization which is parallel to one of magnetic sublattices, the angles between the pairs $\mathbf{M_1},\mathbf{M_3}$, and $\mathbf{M_2},\mathbf{M_3}$ are equal but different from 120°. This phase is stable at $J(T) < 0$, with $\mathbf{M}||\ \mathbf{N_1}\perp\mathbf{N_2}$, $|\mathbf{N_1}| \neq |\mathbf{N_2}|$, $M_{\parallel} = \sqrt{(J-3D'/2)/D}$. The spin symmetry of the phase corresponds to C_2 with the symmetry axis parallel to $\mathbf{M_3}$.

An external magnetic field modifies the phase diagram in a different way, depending on

its orientation with respect to the plane spanned by the vectors $\mathbf{M_1}$, $\mathbf{M_2}$ and $\mathbf{M_3}$. A magnetic field applied parallel to the plane stabilizes configuration FO and contributes to the magnetization, $M_{\parallel}\ (H_{\parallel})$. Typical field dependencies calculated at different values of *J(T)* are shown in Supplementary Fig. 12a.

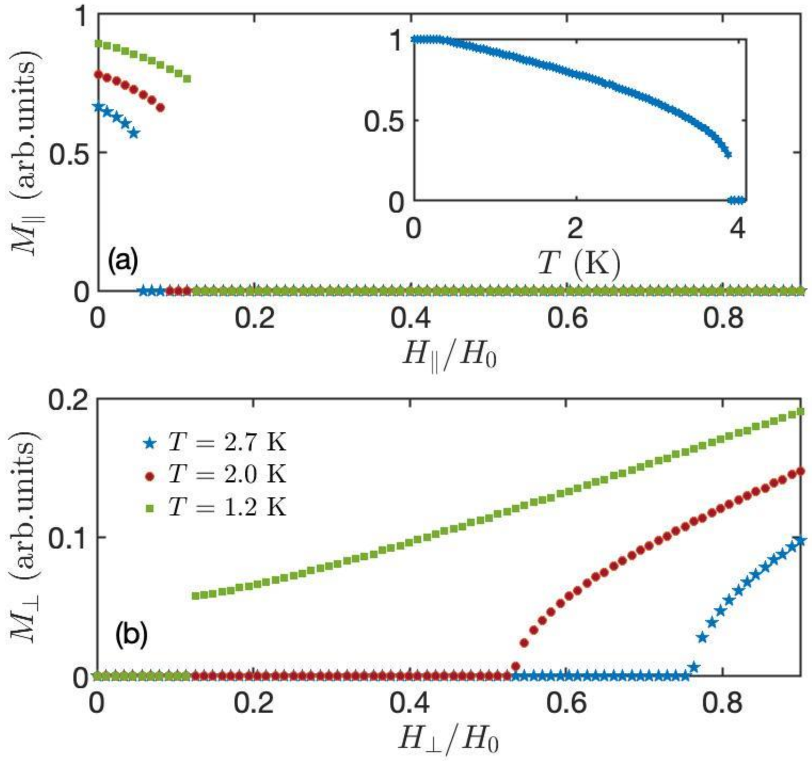

Supplementary Fig. 12 | Magnetization (theory). Typical field dependencies of **a,** in-plane $M_{\parallel}\ (H_{\parallel})$ **b,** out-of-plane $M_{\perp}\ (H_{\perp})$ components of magnetization at different temperatures. Inset in **a** shows temperature dependence of $M_{\parallel}\ (T)$ at zero field. Source data are provided as a Source Data file.

A magnetic field that is perpendicular to the plane induces a transition to the third phase FL with a noncoplanar configuration of the magnetic vectors (Fig. 1f, violet line in Supplementary Fig. 12). In this case, the angles between the vectors $\mathbf{M_1}$, $\mathbf{M_2}$ and $\mathbf{M_3}$ are equal but different from 120°. The magnetization is parallel to the magnetic field, $\mathbf{M}||\mathbf{H} \perp \mathbf{N_1} \perp \mathbf{N_2}$, $|\mathbf{N_1}| = |\mathbf{N_2}| < \sqrt{3}M_s$, $M_{\perp}\ (H_{\perp})$ (see Supplementary Fig. 12b). The stability regions of each of the phases are shown in the Fig. 3 (solid blue and dashed grey lines) of the main text and reproduced in Supplementary Fig. 13 (solid blue and violet lines).

Next, we analyze the structure of the resistivity tensor in each phase based on the spin

symmetry. Corresponding results are summarized in Table 1 of the main text. A small non-diagonal component of the resistivity observed in the experiment is allowed by the low magnetic symmetry of the FO phase. We associate the reduction in symmetry with the rotation of the magnetic moments with respect to the crystallographic axes. Corresponding calculations are given below. We additionally refer the reader to Refs. 3, 4, and 8 where similar effects have been analyzed in detail.

Phase diagram of the polycrystalline sample

To fit the experimental field and temperature dependence of the resistivity, we take into account that our sample is polycrystalline and consists of different grains with arbitrary orientations of the crystallographic axes with respect to the external magnetic field. We divide the averaging over the different crystallites into two steps, taking into account the distribution of (i) the induced in-plane magnetization $M_{\|}$, which determines the value of the magnetic resistivity; (ii) the transverse components of the resistivity with respect to the crystallographic axes.

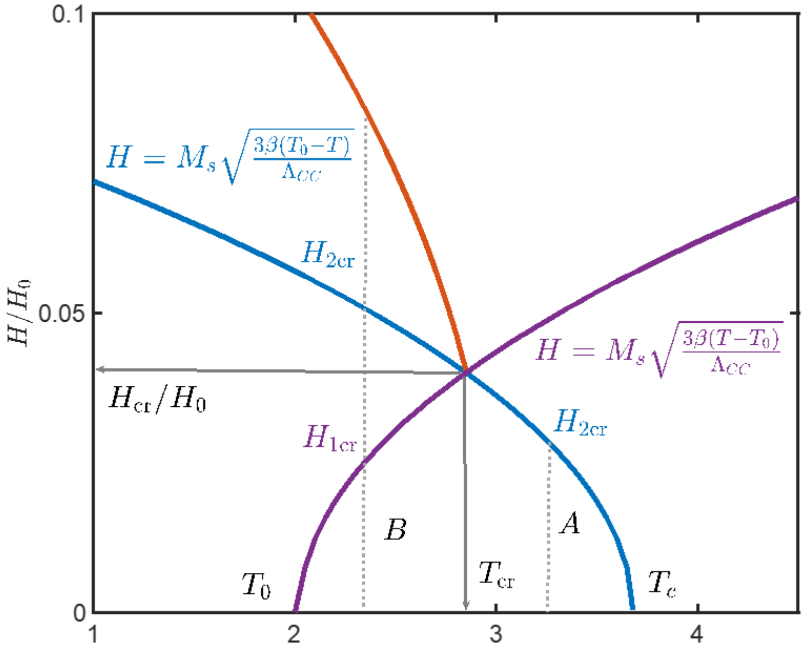

Supplementary Fig. 13 | Temperature-magnetic field phase diagram (theory). The blue line separates the FO and Γ^{5g} phases. Above the violet line the FO and Γ^{5g} phases are unstable with respect to the fluctuation of $\mathbf{M} \perp \mathbf{N_1} \perp \mathbf{N_2}$ (FL phase). The red line shows the transition between the FO and FL phases for a polycrystalline sample (after averaging). Above the tri-critical point $T_{cr} < T < T_0$ the magnetic field stabilizes only the FO phase (dotted line A), all crystallites contribute to the transverse resistivity signal.

Below the critical temperature $T < T_{cr}$ and starting from $H_{1cr} < \mathrm{H}$ (dotted line line B) a polycrystalline sample is a mixture of FO and I phases.

So, in the first step we fix the value of the magnetic field, *H*, and consider different orientations with respect to the sublattice magnetizations. We distinguish only between the diagonal component of the resistivity tensor, as they are introduced in the main. In this case, the distribution of crystallites is parameterized by the angle θ between the magnetic field and the normal to the plane spanned by the vectors $\mathbf{N_1} \perp \mathbf{N_2}$ (hereafter referred to as the ordering plane). Above the tri-critical temperature $T_{cr} < T < T_0$ (see Supplementary Fig. 13, vertical line A), the value of the in-plane magnetization in a particular crystallite is determined by the projection of the magnetic field onto the ordering plane: $M_{\parallel}(\theta) \equiv M_{\parallel}(H\cos\theta)$ [see Eq. (S1)]. The probability to find a crystallite with $M_{\parallel}(\theta)$ is $w(\theta)d\theta = \sin\theta d\theta$ and the average value of the resistivity is calculated as

$$\rho_{ave} = \int \rho[M_{\parallel}(\theta)]w(\theta)d\theta \qquad \text{(S2)}$$

with integration over all $\cos\theta \leq H_{2cr}/H$.

Below the tri-critical temperature $T < T_{cr}$ and above $H_{1cr} < H$ (see Supplementary Fig. 13, vertical line B), the out-of-plane magnetization $M_{\perp}(\theta) \equiv M_{\perp}(H\sin\theta)$ can occur. In this case the value of the in-plane magnetization is given by the following expression:

$$\mathrm{M}_{\parallel}(\theta) = \begin{cases} M_{\parallel}(H\ \cos\theta), & H < H_{1\mathrm{cr}} \\ M_{\parallel}(H\ \cos\theta\sqrt{1 - M^2{}_{\perp}/2}), & H \geq H_{1\mathrm{cr}} \end{cases}, \qquad \text{(S3)}$$

In this case integration in (S2) spans all $cos\theta \leq H_{2cr}/H$ if $H < H_{1\mathrm{cr}}$, and $cos\theta \leq H_{2cr}/(H\sqrt{1 - M^2{}_{\perp}(\theta)/2})$ if $H \geq H_{1\mathrm{cr}}$.

In the second step we start from the expression of resistivity tensor for a single crystallite with account of spin-orbit interactions, as will be explained below. In FO case this symmetric tensor has a nondiagonal component $\rho_{non} \propto \rho_{xx} - \rho_{yy}$, due to rotation of the ordering plane. We next average this tensor over all possible orientations of the crystallographic axes x,y,z. Three dimensional rotations are parametrized with the Gibb's vector or Rodrigues parameters Ref. 9 which gives $\rho_{jk}^{ave} = \frac{1}{5}\rho_{jk} + \frac{4}{5}\delta_{jk}Tr\hat{\rho}$, where δ_{jk} is the Kronecker symbol.

Origin of the transverse component of resistivity

In this section we consider a single crystallite with the ordering plane (111). In this case, the structure of the resistivity tensor is determined by the magnetic symmetry of the phase and depends on the orientation of the magnetic vectors **M**, $\mathbf{N}_1$, and $\mathbf{N}_2$ with respect to the crystallographic axes. By minimizing the exchange energy given by Eq. (1) in the main text we found the values of **M**, $\mathbf{N}_1$ and $\mathbf{N}_2$ as a function of field and temperature. The orientation with respect to the crystallographic axes can be then calculated by minimizing the magnetic anisotropy energy F_{an}. To construct F_{an} we follow the approach described in Refs. 3,4,8 and introduce combinations **Q** and **P** which are irreducible representations of the magnetic symmetry group:

$$\begin{aligned} Q_x &= \tfrac{1}{2}\left(N_{2y} - N_{1x} - \sqrt{2}N_{1z}\right), & P_x &= \tfrac{1}{2}\left(N_{2x} + N_{1y} + \sqrt{2}N_{2z}\right), \\ Q_y &= \tfrac{1}{2}\left(N_{2x} + N_{1y} - \sqrt{2}N_{2z}\right), & P_y &= \tfrac{1}{2}\left(N_{2y} - N_{1x} + \sqrt{2}N_{1z}\right), \\ Q_z &= -\tfrac{1}{\sqrt{2}}\left(N_{2y} + N_{1x}\right), & P_z &= \tfrac{1}{\sqrt{2}}\left(N_{1y} - N_{2x}\right). \end{aligned} \quad \text{(S4)}$$

Here the axes x, y, and z are aligned along $[\bar{1}\bar{1}2]$, $[1\bar{1}0]$ and [111] crystallographic directions. The values of Q, P, and M are not independent due to constraints $|\boldsymbol{M}_1| = |\boldsymbol{M}_2| = |\boldsymbol{M}_3| = M_S$: $\boldsymbol{Q}^2 + \boldsymbol{P}^2 + \boldsymbol{M}^2 = M_S^2$ etc (see Refs. 3,4). The magnetic anisotropy energy which is invariant with respect to the crystallographic symmetry group is modelled as follows:

$$F_{\mathrm{an}} = \tfrac{1}{2}K_{an}\mathbf{Q}^2 + d_{DMI}\mathbf{M}(\mathbf{H})\mathbf{Q}, \quad \text{(S5)}$$

where $K_{an} > 0$ is the anisotropy constant, d_{DMI} can be interpreted as a constant of Dzyaloshinskii-Moriya interactions (it couples magnetization with the antiferromagnetic order parameters). The equilibrium values of **Q** and **P** are calculated by minimizing energy (S1) with the constraints, and the fixed value of $\mathbf{M}(\mathbf{H})$ that minimizes the exchange energy Eq. (S1).

The analysis of the energy Eq. (S5) shows that in the Γ^{5g} phase the only nontrivial component is P_z; in the flower phase only $P_z, Q_z, M_z \neq 0$, and in the FO phase the nonzero components are P_x, P_z and Q_y, M_y. Appearance of the in-plane components P_x, and Q_y corresponds to rotation of the ordering plane away from crystallographic orientation (111).

Symmetry considerations predict the following structure of the resistivity tensor:

$$\rho_{zz} - \rho_{xx}, \rho_{zz} - \rho_{yy} \propto \mathrm{A}\boldsymbol{MQ} + \mathrm{B}\boldsymbol{P}^2, \rho_{xz} \propto P_x P_z \quad \text{(S6)}$$

From Eq. (S6) we conclude that only the FO (and I) phases have a nonzero nondiagonal component of resistivity tensor (see Supplementary Fig. 14).

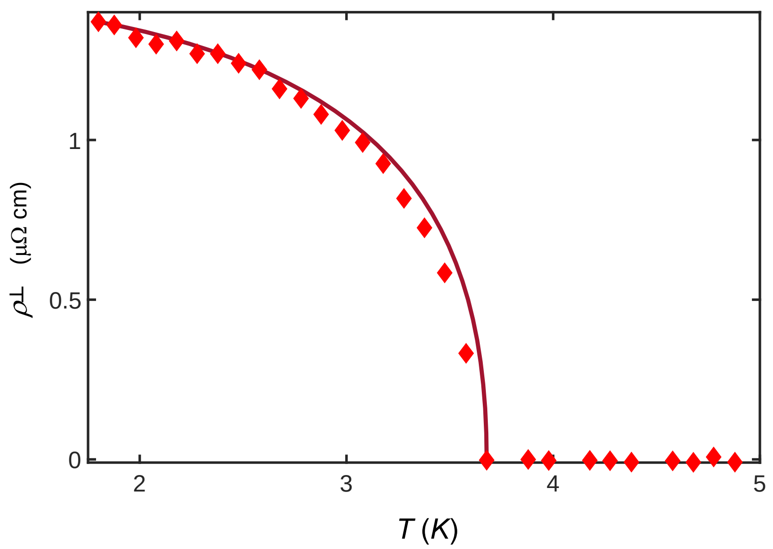

Supplementary Fig. 14 | Temperature dependence of the transverse resistivity in zero magnetic field. Diamonds: $|\rho_\perp|$ from experiment, sample #2, solid line: theory (see text for the details). Source data are provided as a Source Data file.

References

1. Wang, C. et al. Tuning the range, magnitude, and sign of the thermal expansion in intermetallic Mn_3(Zn, M)N (M = Ag, Ge). *Phys. Rev. B* **85**, 220103 (2012).

2. Sun, Y. et al. Lattice contraction and magnetic and electronic transport properties of $Mn_3Zn_{1-x}Ge_xN$. *Appl. Phys. Lett.* **91**, 231913 (2007).

3. Gomonaj, E. V. & L'vov, V. A. A theory of spin reorientation and piezomagnetic effect in noncollinear Mn_3AgN antiferromagnet. *Phase Transitions* **40**, 225-237 (1992).

4. Gomonaj, E. V. & L'vov. V. A. Phenomenologic study of phase transitions in noncollinear antiferromagnet of metallic perovskite type. *Phase Transitions* **38**, 15-31 (1992).

5. Grzybowski, M. J. et al. Antiferromagnetic Hysteresis above the Spin Flop Field. *Phys. Rev. B* **107**, L060403 (2023).

6. Grytsiuk, S. et al. Topological–chiral magnetic interactions driven by emergent orbital magnetism, *Nature Comm.* **11**, 511 (2020).

7. Gomonay, O. Berry-phase effects and electronic dynamics in a noncollinear antiferromagnetic texture. *Phys. Rev. B* **91**, 144421. (2015)

8. Rajan, A. et al. Revealing the higher-order spin nature of the Hall effect in non-collinear antiferromagnet $Mn_3Ni_{0.35}Cu_{0.65}N$ https://arxiv.org/abs/2304.10747

9. Rodrigues, O. Des lois géometriques qui regissent les déplacements d'un systéme solide etc. *J. Math. Pures Appl.* **5**, 380 (1840)
